# Individual odour signatures that mice learn are shaped by involatile major urinary proteins (MUPs)

**DOI:** 10.1186/s12915-018-0512-9

**Published:** 2018-04-27

**Authors:** Sarah A. Roberts, Mark C. Prescott, Amanda J. Davidson, Lynn McLean, Robert J. Beynon, Jane L. Hurst

**Affiliations:** 1 Mammalian Behaviour & Evolution Group, Institute of Integrative Biology, University of Liverpool, Leahurst Campus, Neston, CH64 7TE UK; 2 Centre for Proteome Research, Institute of Integrative Biology, University of Liverpool, Crown Street, Liverpool, L69 7ZB UK

**Keywords:** Individual recognition, Olfactory communication, Communication signals, Signature mixtures, Pheromones, House mice, MUPs, MHC, Scent marks, Darcin

## Abstract

**Background:**

Reliable recognition of individuals requires phenotypic identity signatures that are both individually distinctive and appropriately stable over time. Individual-specific vocalisations or visual patterning are well documented among birds and some mammals, whilst odours play a key role in social recognition across many vertebrates and invertebrates. Less well understood, though, is whether individuals are recognised through variation in cues that arise incidentally from a wide variety of genetic and non-genetic differences between individuals, or whether animals evolve distinctive polymorphic signals to advertise identity reliably. As a bioassay to understand the derivation of individual-specific odour signatures, we use female attraction to the individual odours of male house mice (*Mus musculus domesticus*), learned on contact with a male’s scent marks.

**Results:**

Learned volatile odour signatures are determined predominantly by individual differences in involatile major urinary protein (MUP) signatures, a specialised set of communication proteins that mice secrete in their urine. Recognition of odour signatures in genetically distinct mice depended on differences in individual MUP genotype. Direct manipulation using recombinant MUPs confirmed predictable changes in volatile signature recognition according to the degree of matching between MUP profiles and the learned urine template. Both the relative amount of the male-specific MUP pheromone darcin, which induces odour learning, and other MUP isoforms influenced learned odour signatures. By contrast, odour recognition was not significantly influenced by individual major histocompatibility complex genotype. MUP profiles shape volatile odour signatures through isoform-specific differences in binding and release of urinary volatiles from scent deposits, such that volatile signatures were recognised from the urinary protein fraction alone. Manipulation using recombinant MUPs led to quantitative changes in the release of known MUP ligands from scent deposits, with MUP-specific and volatile-specific effects.

**Conclusions:**

Despite assumptions that many genes contribute to odours that can be used to recognise individuals, mice have evolved a polymorphic combinatorial MUP signature that shapes distinctive volatile signatures in their scent. Such specific signals may be more prevalent within complex body odours than previously realised, contributing to the evolution of phenotypic diversity within species. However, differences in selection may also result in species-specific constraints on the ability to recognise individuals through complex body scents.

**Electronic supplementary material:**

The online version of this article (10.1186/s12915-018-0512-9) contains supplementary material, which is available to authorized users.

## Background

The ability to identify individual conspecifics reliably allows animals to adjust their social responses according to information gained from previous encounters with those individuals [1, 2]. For reliable discrimination, signatures must be sufficiently polymorphic at an individual level and consistent over a timeframe appropriate to recognition [3]. There is ample evidence that such individual-specific signatures are widespread. Individual conspecifics are recognised, for example, through vocalisations in many bird, anuran and mammal species [4–6], by visual facial features in primates [7, 8] and some paper wasps [9], or through odour differences in many mammals [10, 11] and insects [12]. Less well understood is the extent to which such polymorphic signatures evolve to provide individually distinctive signals that advertise the signaller’s identity, because of benefits to the signaller derived from being recognised. Instead, receivers may make use of cues that vary inadvertently between individuals and allow identification but have not evolved as signals to facilitate recognition [2, 13]. Understanding how distinctiveness arises in the features used for recognition is essential to establish whether animals use evolved signals or incidental cues.

Animal scents are characterised by considerable molecular complexity, potentially communicating a wide range of information about both the current metabolic and social status of a scent owner and its identity [14, 15]. Volatile body odours, arising from scent glands and excreta, are influenced by complex interactions between the animal’s genotype and its current physiological status, infection status, diet, microbiome and freshness of the scent [16]. As a result, there is much variation in odour composition within as well as between individuals, typically involving a wide range of volatile organic compounds (VOCs) [17]. To reliably identify the owner, animals need to discriminate specific components that are expressed consistently by the same individual. Components that reflect genetically ‘fixed’ differences between individuals provide the most likely candidates [2]. Research on inbred rodents kept under standardised laboratory conditions suggests that many genes contribute to discriminable differences in odour, with attention focused particularly on genes of the major histocompatibility complex (MHC) as one of the most individually variable regions of the vertebrate genome. These genetically determined body odours, termed ‘odortypes’, have been assumed to provide the odour signatures used for individual recognition [18, 19]. The implication is that scent recognition signatures (also called chemical signature mixtures [20]) arise incidentally from the very large number of genetic differences between individuals in natural outbred populations. These differences can influence scent, including the highly variable MHC, without a need for signallers to evolve distinctive identity signals. This has led to the speculation that chemical signature mixtures in general are cues used by receivers to identify others rather than evolved signals of identity [20].

In contrast to studies of inbred laboratory mice, studies of genetically heterogeneous wild house mice (*Mus musculus domesticus*) indicate that mice have evolved a set of polymorphic proteins, specialised for scent communication, that provide a distinctive identity signal in their urinary scent marks. These proteins, called major urinary proteins (MUPs), are released at high concentration in mouse urine, encoded by a cluster of more than 20 functional genes on mouse chromosome 4 [21, 22]. Individual signatures arise largely through a combination of variation in sequence and differential transcription of genes located in the central region of the *Mup* cluster (central MUPs), such that individuals express consistent but different patterns of MUP isoforms [21, 23]. Balancing selection acts on both the frequencies of central MUP variants in the population and on differential transcription to maintain the combinatorial diversity of variants between individuals [23]. Notably, MUP signatures appear to drive recognition of the owners of urinary scent marks, regardless of other genetic or non-genetic differences [24–27]. These signatures can be discriminated on nasal contact through a set of vomeronasal sensory neurons that use a combinatorial-coding strategy sensitive to different ratios of MUP isoforms in a mixture [27].

MUPs have a high degree of structural stability that confers resistance to degradation in the environment [28]. However, a requirement to contact and transport non-volatile proteins to the vomeronasal organ for detection means that gaining information is relatively slow and brings the risk of attack whilst animals are in such close proximity [29, 30]. Airborne odours detected through the main olfactory system allow much faster recognition at a distance without contact [31], and animals are known to learn volatile odour signatures of familiar individuals that have biological significance to them (such as mates, familiar social companions or competitors). Contact with an atypical MUP sex pheromone darcin (MUP20) in male house mouse urine induces females to rapidly learn attraction to the male’s volatile odour signature and to the spatial location of the darcin pheromone through associative learning [32, 33]. This learned attraction to a male’s volatile signature is retained for several weeks after initial contact investigation [30]. Although a male’s volatile odour signature and involatile MUP signature involve completely distinct categories of molecules that are derived independently, these different types of molecular signature may be physically linked. Structurally, MUPs enclose a central cavity that binds low molecular weight (LMW) hydrophobic molecules and slows their release from scent marks [34–37], sequestering a wide range and quantity of VOCs in mouse urine [38]. Differences in the residues that line the MUP cavity determine its size, shape and tightness of binding ligands, resulting in isoform-specific differences in ligand selectivity and retention [36, 39–41]. However, the extent to which the profile of different MUPs expressed in urine influences a mouse’s volatile odour signature is unknown.

Here, we investigate the influence of the MUP profile on the volatile odour signatures that mice learn from scent marks. After finding that learned volatile odour signatures are bound and released by urinary proteins, we manipulated MUP profiles to establish the extent to which MUPs (1) influence behavioural recognition of individual volatile signatures and (2) alter the profile of specific male signalling volatiles emanating from scent. We show that the genetically determined profile of MUPs in a mouse’s urine plays a dominant role in determining learned odour signatures. Distinctive odour signatures depended on differences in individual-specific MUP profiles, and recognition changed predictably when MUP profiles were directly manipulated. VOCs not bound to urinary proteins had no influence on volatile signature recognition. Further, manipulation of MUP profiles led to MUP-specific and volatile-specific changes in the airborne release of volatile ligands from scent deposits. The odour signatures learned and used by mice to recognise individuals are not cues that arise incidentally from the large number of genetic differences between individuals. Instead, they are very strongly shaped by a specific set of polymorphic communication proteins that has evolved to provide a distinctive signal of identity.

## Results

### Behavioural recognition

#### Individual airborne odour signatures are bound by urinary proteins

**Fig. 1:**
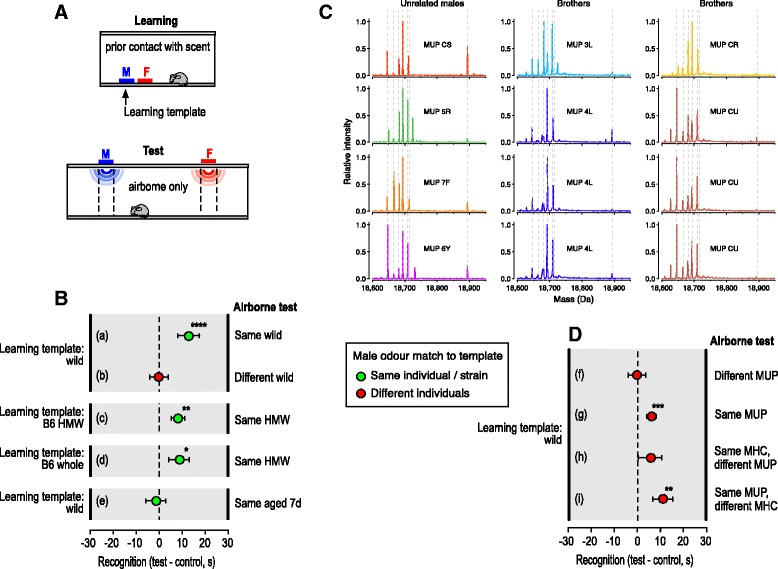
Individual airborne odour signatures correlate with MUP phenotype. **A** Bioassay of individual airborne signature recognition. After contact with 10 μl of male urine (learned template) and 10 μl of female urine (control) for 30 min, female wild-stock mice were tested with airborne odour in a different arena. **B**, **D** Difference in time under the male test compared to female control stimulus (mean ± standard error of the mean (SEM)). Recognition was assessed as more time near the male test stimulus than the female control, using matched pair *t* tests of log transformed data ((a) *t*_11_ = 4.06, *p* = 0.0009; (b) *t*_11_ = − 0.18, *p* = 0.57; (c) *t*_11_ = 2.95, *p* = 0.007; (d) *t*_11_ = 2.54, *p* = 0.014; (e) *t*_11_ = − 1.13, *p* = 0.86; (g) *t*_19_ = 2.89, *p* = 0.0047; (h) *t*_19_ = 1.38, *p* = 0.092) or non-parametric Wilcoxon signed rank tests ((f) *z* = − 0.68, *p* = 0.74, *n* = 15; (i) *z* = 2.46, *p* = 0.006, *n* = 20). **C** Examples of typical MUP patterns revealed by ESI-MS of urine from wild-stock males for four unrelated males (*left*) or two sets of four brothers (*numbers* or *letters* denote MUP haplotypes). The intensity of each mass peak is expressed relative to the highest peak in the spectrum. Brothers of the same MUP type (same colour) have extremely similar profiles. *Dashed lines* indicate masses predicted by one or more central *Mup* genes present in the B6 reference genome (masses 18,645, 18,665, 18,682, 18,694, 18,708, 18,713 Da) or darcin (18,893 Da), though only a subset of these is expressed in common laboratory strains (see Fig. 2). *p < 0.05, **p < 0.01, ***p < 0.005, ****p < 0.001

To assess recognition of individual airborne odour signatures, we used female learned attraction to the airborne odour signature of a male, induced when female mice contact darcin in a male’s urine [30, 32, 42]. This functional test of learned attraction to a specific individual’s odour overcomes the lack of specificity of simple odour discrimination tasks, in which any discriminable difference between odours stimulates a response without implying a role in individual recognition [1]. Subjects were genetically heterogeneous wild-stock female mice that were unfamiliar with, and unrelated to, odour donors. To learn a male’s odour signature, females were placed individually in clean cages for 30 min where they could contact 10 μl of a male urine stimulus (the learned scent template) and 10 μl of a female urine stimulus that differed from the subject’s own scent and acted as a control novel urine stimulus (see section on Behavioural recognition assay in Methods). Females were then tested in a different clean arena with male and control female urine stimuli presented above a perforated lid that prevented direct contact (Fig. 1A).

We confirmed that this bioassay successfully assessed recognition of individual airborne odour signatures: when airborne urinary odour came from the same wild-stock male donor as the learned urine template, females were more attracted to the male than to the control female odour (Fig. 1B (a)). No such attraction was shown towards odour from a different male, unrelated to the learned scent template donor (Fig. 1B (b)).

Next, we tested whether airborne odour signatures are associated with the high molecular weight (HMW) fraction of urine that is retained by a 3 kDa filter. This fraction contains involatile urinary proteins together with any LMW ligands bound to these proteins, and has a strong mousey odour to the human nose. Females were attracted to odour from the HMW fraction of male urine after contact with this fraction as the learning template (Fig. 1B (c)). They were similarly attracted to odour from the HMW fraction after contact with whole male urine that contains all urinary components (Fig. 1B (d)). This suggests that the learned odour signature consists of volatiles bound and released by urinary proteins in the HMW fraction. To eliminate any possibility that females could detect involatile proteins in the airborne stimulus, we confirmed that there was no attraction when urine had been air-dried for 7 days to deplete the volatile ligands (Fig. 1B (e)); the characteristic mousey odour is no longer apparent after this time, but MUPs persist without significant degradation. Thus, the airborne signature that females recognise comprises VOCs that are bound and released by urinary proteins, not the proteins themselves.

#### MUP but not MHC polymorphism correlates with learned odour signatures

The HMW protein fraction of adult mouse urine consists largely of MUPs and their hydrophobic LMW ligands [38, 43]. Individual-specific MUP signatures are encoded by a set of highly similar paralogues in the central region of the MUP gene cluster [21, 44], which show differential transcription across individuals [23]. As MUP isoforms can differ in their ligand binding affinities [36, 39–41], we hypothesised that MUP signatures might influence volatile signatures. Unrelated individuals normally express distinct MUP signatures [23, 45] (Fig. 1C), but close relatives that inherit the same MUP type express remarkably similar profiles of central MUPs, even on the heterogeneous background of outbred wild-stock mice (Fig. 1C, see also [46]). To test the influence of shared MUP signature on volatile signatures, we examined the extent to which recognition generalised between odours from two male sibs according to their sharing of MUP genotype on a randomly assorting genetic background. Females that learned the odour signature of one wild-stock male generalised their attraction to odour from a male’s sib that shared the same MUP type as the learned template (Fig. 1D (g); *t*_19_ = 2.89, *p* = 0.0047) but not to a sib of different MUP type (Fig. 1D (f); *z* = − 0.69, *p* = 0.74; bias in response to test minus control odour when MUP type was shared or different: Mann-Whitney U test, *z* = 2.13, *p* = 0.033). Thus, females recognised similarity in the odour signatures of males with the same MUP type, despite many random genetic differences between outbred wild-stock males.

It has been suggested that the highly polymorphic MHC is the main source of individually distinctive urinary odours in mice [19], although the mechanism by which MHC influences VOCs in urine remains elusive [47, 48]. Recognition of odour signatures did not generalise consistently to odour from a male’s sib with the same MHC type as the learned template donor (*t*_19_ = 1.38, *p* = 0.09), although it appeared that a few females may have recognised this odour (Fig. 1D (h)). However, the lack of statistically significant recognition contrasted with the reliable generalisation seen across odours from males of the same MUP type, even when these donors were selected to have different MHC types (Fig. 1D (i); *z* = 2.46, *p* = 0.006). Thus, MUP type has a dominant influence on the male’s volatile odour signature that females learn in genetically heterogeneous wild-stock mice. The variable response to shared MHC type is consistent with findings that volatiles associated with MHC type are strongly influenced by interaction with background genes and environmental influences [47–49].

As other differences between males (genetic and non-genetic) are likely to influence the cocktail of VOCs available to bind to urinary MUPs, we expected the most consistent recognition when the test odour came from the same individual as the learned scent template. To compare the distribution of responses, we ran an additional set of trials to assess response to odour from the same individual as the learned template. However, the distribution of attraction responses when odour came from the same individual (bias in time near male minus female control stimulus: 12.9 ± 2.6 s, *n* = 31) did not differ significantly from that towards a sib male of the same MUP type (Fig. 1D (g, i) combined: 8.5 ± 2.5 s, *n* = 40; Kolmogorov-Smirnov *z* = 1.01, *p* = 0.26). By contrast, attraction clearly differed when odour came from the same individual versus a sib of different MUP type (Fig. 1D (f, h) combined: 2.5 ± 3.2 s, *n* = 37; *z* = 1.93, *p* = 0.001). Even with such a large sample size, females failed to show any evidence of recognition if the male’s MUP type differed from that of the template learned (*n* = 37, *z* = − 0.42, *p* = 0.66). MUP type clearly has a surprisingly strong influence on the odour signature that females learn.

#### Darcin concentration influences individual odour signatures

Learned attraction to male urinary odour signatures is induced by contact with male-specific MUP20, known as darcin [32]. Whilst all normal adult male house mice express this pheromone in their urine, males differ in the amount expressed relative to other MUPs (Fig. 1C; see also [50, 51]). Darcin has greater sequence divergence from other MUPs compared to the very limited divergence found between central MUPs, and the size and shape of its cavity confers substantial differences in binding affinity for hydrophobic volatile ligands [28, 40, 41]. To test whether differences in the amount of darcin in male urine significantly influence a male’s volatile signature, we manipulated this using recombinant protein (r-darcin). To standardise the odour stimuli in these tests, we used urine from three laboratory mouse strains that differ in the amount of darcin expressed, but have similar central MUP signatures [21, 52].

**Fig. 2:**
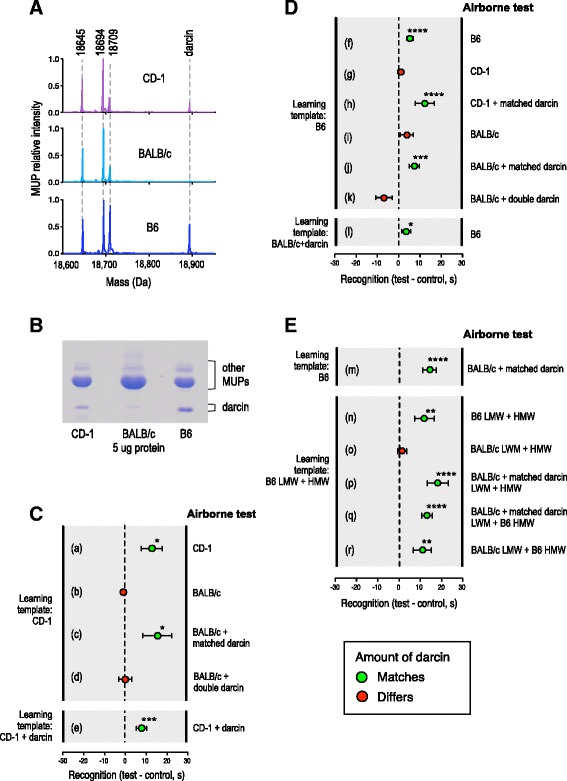
Darcin concentration influences individual volatile signature recognition. Levels of darcin were manipulated in male urine from three laboratory mouse strains to investigate the influence on volatile signature recognition. **A** ESI-MS of male urine from the three different strains (CD-1, known also as ICR; BALB/c; B6: C57BL/6 J). *Dashed lines* show the correspondence of mass peaks across all three strains. **B** SDS-PAGE confirmed different levels of darcin in the three strains. **C**, **D** Recognition of airborne odour within and between strains when r-darcin was added to match (c, e, h, j, l) or not match (d, k) the amount of darcin in the learned template. Buffer was added to all learned template and airborne test stimuli as a control. **E** Recognition of airborne odour when LMW and HMW urine fractions were mixed within or between strains and the amount of darcin in the HMW fraction matched (m, n, p, q, r) or did not match (o) that in the learned template. Data in **C**–**E** show the difference in time under the male test odour compared to female control (mean ± SEM). Recognition was assessed as more time near the male test stimulus than the female control, using matched pair *t* tests of untransformed ((a) *t*_11_ = 2.56, *p* = 0.013; (e) *t*_19_ = 3.25, *p* = 0.002; (m) *t*_11_ = 4.48, *p* = 0.0005; (o) *t*_11_ = 0.76, *p* = 0.23) or log transformed data ((c) *t*_11_ = 2.53, *p* = 0.014; (d) *t*_11_ = 0.38, *p* = 0.36; (f) *t*_12_ = 4.86, *p* = 0.0002; (h) *t*_11_ = 4.13, *p* = 0.0008; (i) *t*_19_ = 1.10, *p* = 0.14; (k) *t*_11_ = − 1.21, *p* = 0.87; (l) *t*_16_ = 1.83, *p* = 0.043; (n) *t*_11_ = 2.89, *p* = 0.007; (p) *t*_11_ = 4.12, *p* = 0.0008; (q) *t*_11_ = 6.88, *p* < 0.0001), or non-parametric Wilcoxon signed rank tests ((b) *z* = − 0.77, *p* = 0.75, *n* = 12; (g) *z* = 0.56, *p* = 0.30, *n* = 20; (j) *z* = 2.72, *p* = 0.002, *n* = 18; (r) *z* = 2.31, *p* = 0.009). *p < 0.05, **p < 0.01, ***p < 0.005, ****p < 0.001

CD-1 (Swiss ICR lineage) and most substrains of BALB/c mice (Castle lineage) express very similar profiles of central MUPs, with all individuals expressing the same pattern [52] (Fig. 2A). However, darcin comprises approximately 8% of total urinary MUP in CD-1 males but is expressed at only trace levels in BALB/c (< 0.5% total MUP [32], Fig. 2B). After contact with CD-1 male urine as a learning template, female attraction generalised to odour from other CD-1 males (Fig. 2C (a)), but not to BALB/c male odour (Fig. 2C (b)), despite shared central MUP profiles. Attraction generalised across strains, though, when r-darcin was added to the BALB/c male stimulus to match the amount in the CD-1 learning template (Fig. 2C (c)). There was no attraction if double the amount of darcin was added to the BALB/c male test odour (Fig. 2C (d)), although double the amount of darcin resulted in attraction when this matched the amount in the learning template (Fig. 2C (e)). This suggests that a matched amount of darcin is critical for odour recognition, not simply its presence or absence.

To test this further, as another learning template we used urine from C57BL/6 reference strain males (hereafter B6), which express a higher level of darcin (approximately 16% of their total urinary MUP, Fig. 2B). As predicted, females generalised attraction to urinary odour from other B6 males (Fig. 2D (f)), but not to odour from CD-1 or BALB/c males unless r-darcin was added to the test odour at an amount that matched the B6 learning template (Fig. 2D (g–l)). Thus, the amount of darcin in male urine critically influences the distinctiveness of male odour signatures that females learn. Other minor differences in central MUPs expressed by B6 compared to the other two strains did not prevent generalisation between their odours once the amount of darcin was equalised: two different charge variants are present in the 18,694 Da mass peak in BALB/c but only one in B6 [32, 53], whilst the 18,709 Da central isoform differs by a single amino acid between strains (Q_136_K mature sequence numbering [53]). These differences are all on the surface of MUPs rather than within the central cavity that binds volatiles and do not appear to have a major effect on volatile signatures.

To further understand the impact of darcin on individual odour signatures, we conducted a more extreme manipulation of the protein-bound and unbound urinary volatiles between B6 and BALB/c mouse strains. These two strains exhibit multiple quantitative differences in overall urinary volatile profiles [47, 49]. First, we reconfirmed that females generalise between odour learned on contact with unmanipulated B6 male urine and odour from BALB/c male urine with r-darcin added to match that in the learned template (Fig. 2E (m)). We then fractionated male urine into HMW components retained by a 3 kDa filter (proteins and bound LMW ligands) and LMW components not bound to proteins. This allowed us to mix together the unbound LMW components of urine with protein and protein-associated components from the same or different strains. Recombined HMW and LMW fractions of B6 male urine provided the learning template in each test (Fig. 2E). When test urine consisted of HMW and LMW from the same strain, responses were very similar to equivalent tests with intact urine, confirming that the fractionation and recombination process did not interfere with learned recognition. Females were attracted to odour from another B6 HMW and LMW recombined pool that matched the learning template (positive control: Fig. 2E (n)), but were not attracted to BALB/c HMW and LMW recombined (negative control: Fig. 2E (o)) unless matching darcin had been added to the BALB/c male urine prior to fractionation and recombination (Fig. 2E (p)). When BALB/c LMW was mixed with B6 HMW, females were attracted, whether or not darcin had been added to BALB/c urine prior to fractionation (Fig. 2E (q, r)). Indeed, there was no difference in the strength of attraction towards stimuli containing B6 HMW, whether this was combined with LMW from B6, BALB/c, or BALB/c urine with a matched amount of r-darcin (Kruskal-Wallis test, chi-squared = 0.54, *p* = 0.76; Fig. 2E (n, q, r)). Thus, unbound LMW components that differed between the two strains had no influence on volatile signature recognition.

**Fig. 3:**
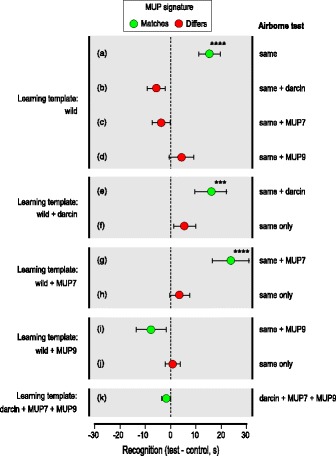
MUP manipulation disrupts recognition of individual volatile signatures from wild-stock males. Urine from wild-stock males was manipulated by the addition of r-darcin, r-MUP7 or r-MUP9 (1 μg/μl) to establish whether this disrupted recognition of the airborne odour signature in the learned template. Data show the difference in time under the male test compared to female control stimulus (mean ± SEM) when the male airborne test stimulus matched the learning template or differed. Recognition was assessed as more time near the male test odour than the female control, using matched pair *t* tests of log transformed data ((a) *t*_11_ = 4.36, *p* = 0.0006; (b) *t*_11_ = − 1.58, *p* = 0.93; (c) *t*_11_ = − 0.48, *p* = 0.68; (d) *t*_11_ = 0.05, *p* = 0.48; (f) *t*_11_ = 0.07, *p* = 0.47; (k) *t*_11_ = − 0.24, *p* = 0.59) or non-parametric Wilcoxon signed rank tests ((e) *z* = − 2.51, *p* = 0.0045; (g) *z* = 2.98, *p* < 0.001; (h) *z* = 0.78, *p* = 0.24; (i) *z* = − 1.18, *p* = 0.87; (j) *z* = − 0.47, *p* = 0.66; *n* = 12 for all tests). A control test (k) examined response to a mixture of all three recombinant MUPs (1 μg/μl of each) when presented as the contacted learning template and matching airborne test odour. ***p < 0.005, ****p < 0.001

As most laboratory mouse strains derive from an extremely limited founder pool [52, 54] and are homozygous, we next manipulated MUP profiles of wild-stock urine donors to understand the impact on odour signatures within the context of normal phenotypic complexity. Females were each tested with urine from a different male donor to establish the general effect of manipulation across multiple random genomes and MUP types. Confirming our findings using laboratory strain donors, the odour signatures of wild-stock males depended on the amount of darcin in their urine. Whilst females were reliably attracted to urine odour from the same male as the learning template (Fig. 3a), this was disrupted by the addition of r-darcin (1 μg/μl) to either the test odour (Fig. 3b) or the learning template (Fig. 3f) unless the same amount was added to both (Fig. 3e).

#### Central urinary MUPs also influence individual odour signatures

Most MUP signature variation between individual house mice involves differential expression of central MUP isoforms [23]. These are highly similar MUPs with amino acid differences located largely on the protein surface. Cavity residues are conserved except for variation between valine and phenylalanine at residue 56, a change that alters ligand binding affinities [36, 39, 41]. To investigate the influence on male odour signatures, we manipulated the amount of two common central MUPs that differ at this cavity residue in wild-stock male urine. MUP7 (mass 18,645 Da) and MUP9 (mass 18,694 Da) are identical at 160 out of 162 amino acids (99% identical), differing only in that MUP7 has valine instead of phenylalanine in the central cavity (V_56_F) and lysine instead of glutamic acid at the surface residue 140 (K_140_E). Expression of MUP7 is very strongly male-biased, whilst MUP9 is expressed by both sexes albeit at greater levels in males [21, 44, 50].

Recognition and attraction to male odour was disrupted by the addition of r-MUP7 (1 μg/μl) to the test odour stimulus (Fig. 3c) or to the contacted learning template (Fig. 3h), unless the same amount was added to both (Fig. 3g). Altering the amount of r-MUP9 (1 μg/μl) between learning template and test odour also appeared to disrupt recognition of a male’s odour signature (Fig. 3d, j). However, in this case, no attraction was evident when a matched amount was added to both (Fig. 3i). This differed from all other tests where the test odour and learning template matched (Figs. 1B (a, c); 2C (a, e), d (f); and 3a, e, g; see also [30, 32]), and suggests that the addition of r-MUP9 disrupted odour learning itself when females contacted the manipulated learning template. Notably, this was the only test that manipulated a urinary component that was not very strongly male-biased in expression. Increasing the amount of MUP9, which has the same cavity residues as all central MUPs in the female control stimulus, reduced the potential difference in MUP ligand binding between male and control female stimuli, even though male-specific components were still present in the male stimulus. This may have reduced the attractiveness of the male stimulus and thus reduced learning during direct contact, or made it harder to discriminate between male and female odours during the test.

Thus, varying the amount of the male-specific MUPs darcin or central MUP7 alters the male odour signature learned by females. To eliminate the possibility that this was due to either (1) volatile contaminants introduced with the purified recombinant MUP from *Escherichia coli* (no contamination was detectable through gas chromatography-mass spectrometry (GC-MS) analysis, but mouse noses could be more sensitive), or (2) detection of r-MUPs themselves in the airborne test stimulus, females were tested with a mixture of the three r-MUPs presented without male urine, after contact with the matching stimulus as the learning template. This failed to stimulate any learned attraction (Fig. 3k), confirming that females did not learn attraction to putative volatile contaminants and did not detect airborne r-MUPs. Instead, responses are consistent with the hypothesis that the amounts of male-specific darcin and MUP7 in urine determine the equilibrium of bound volatiles involved in recognition and their rates of release, and thus the odour signatures that females learn on contact with male urine.

### MUP influence on urinary volatiles

#### Correspondence between MUP and urinary volatile phenotypes

As the specific composition of urinary MUPs plays such a major role in determining the volatile signatures that mice learn, we examined the correspondence between individual MUP type and the profile of airborne volatiles emitted from urine streaks. To match our behavioural tests, we used wild-stock male donors housed individually under standardised laboratory conditions from weaning. To control for relatedness across the genome and any effects of shared rearing in the same litter, we compared urine samples from littermate sibs (coefficient of relatedness, *r* = 0.5) and from double cousins (*r* = 0.25, no parent shared) that inherited the same or different MUP types through recent common descent (see Methods).

**Fig. 4:**
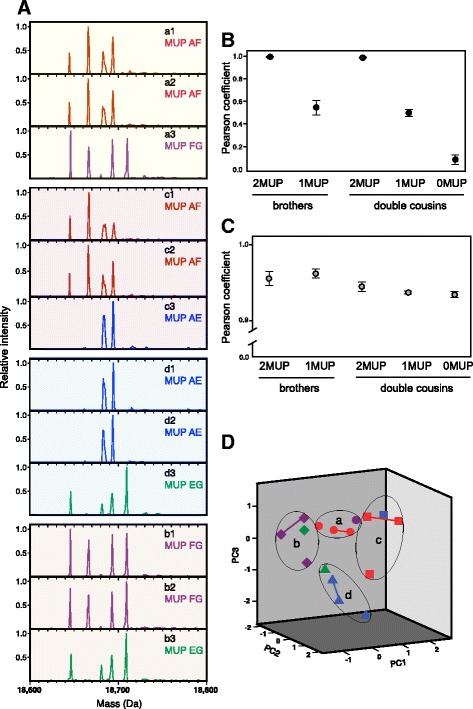
Total urinary volatile phenotypes do not correspond with MUP genotype. **A** ESI-MS intact mass profiles compare central MUP profiles among trios of males in four sib groups (a−d, *background shading*), where sib groups were double cousins (different parents, shared grandparents; *r* = 0.25). Within sib trios (*r* = 0.5), two brothers shared both MUP haplotypes, whilst a third shared only one haplotype with sibs (haplotypes denoted by *capital letters*). **B** Pearson correlation coefficients compare similarity of MUP profiles between each pair of males according to shared MUP haplotypes and level of relatedness, based on the relative proportion of each of seven mass peaks in their intact mass profiles (data are means ± SEM). Each mass peak corresponds to known mouse MUP sequences (see Data analysis section in Methods). **C** Pearson correlation coefficients also compare the similarity of total urinary volatile profiles between pairs of males, based on the areas of 134 peaks extracted by HS-SPME and GC-MS analysis, expressed as proportions of the total peak area in that sample (data are means ± SEM). **D** Principal component analysis of urinary volatile phenotypes reveals clustering of males within sib groups (symbols) based on the first three derived components (explaining 24.6%, 12.7% and 9.5% variance respectively). Two urine samples were analysed from male 1 of each sib group (symbols linked by lines) to compare similarity within and between males. There was no clear clustering according to MUP genotype (colours)

Electrospray ionisation mass spectrometry (ESI-MS) of intact urine samples confirmed that central MUP phenotypes were very similar among sibs and double cousins that inherited the same MUP genotype (both haplotypes shared), each male expressing the same set of mass peaks at very similar relative intensities (Fig. 4A). There was extremely high correlation between central MUP profiles of males sharing the same MUP genotype (*r* = 0.99 ± 0.003), regardless of whether males were littermate sibs or double cousins (*t*_11_ = − 0.45, *p* = 0.66). This similarity was substantially greater than that between males of different MUP types (*r* = 0.37 ± 0.036; *t*_52.8_ = − 17.1, *p* < 0.0001; Fig. 4B).

By contrast, the total pattern of urinary VOCs released from urine streaks, sampled using headspace solid phase micro-extraction (HS-SPME), did not differ between males of the same or different MUP type. Urinary volatile profiles were much more complex than MUP profiles, with 134 different peaks separated by gas chromatography across male samples (excluding contaminants present in controls; volatile peak areas for each sample are given in Additional file 1). Correlation coefficients, based on the relative areas of peaks within each sample, revealed high correlation between all samples (range 0.89–0.99), due to consistent high abundance of some peaks and much lower levels of others. Pairwise comparisons revealed no greater correlation in total volatile profile between males of the same versus different MUP type (*F*_1,80_ = 0.01, *p* = 0.92), although correlation coefficients suggested that sibs had more similar volatile profiles than double cousins with the same level of MUP sharing (*F*_1,80_ = 11.51, *p* = 0.001; Fig. 4C). Principal component analysis confirmed that total urinary volatile profiles clustered according to sib group but not MUP type (Fig. 4D). Thus, although females learn volatile signatures that correlate strongly with the male’s MUP type, we did not detect an effect of MUP type on the overall profile of urinary volatiles. This suggests that the volatile signature learned is selective towards components that are specifically associated with the MUP profile.

**Fig. 5:**
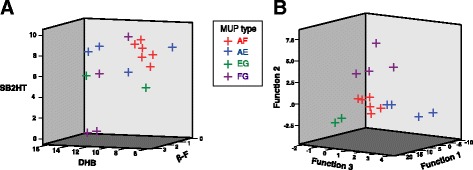
Clustering of MUP types based on volatile profiles. **A** Urine samples from wild-stock males that shared the MUP type AF clustered according to the amount of SB2HT, DHB and βF (expressed as percentages of the total peak area of volatiles in each sample), whilst other MUP types did not cluster based solely on these three volatiles (see Fig. 4 for further details). αF was not detectable in these samples. **B** Discriminant function analysis based on SB2HT, DHB and βF together with five additional volatile peaks (see Table 1) derived three functions that resolved all four MUP types

Next, we explored whether these MUP types differed according to the relative levels of four volatiles that are known MUP ligands [34, 37, 55, 56] and also male pheromones with established reproductive priming effects on female mice [57–59]: 2-*sec*-butyl-4,5-dihydrothiazole (SB2HT), (*R,R*)-3,4-dehydro-*exo*-brevicomin (DHB), *E*-β-farnesene (βF) and *E,E*-α-farnesene (αF). All samples with MUP type AF clustered together due to strong consistency in their proportions of SB2HT and DHB, but these volatiles alone were not sufficient to cluster the other three MUP types in our sample (Fig. 5A). We therefore used a stepwise discriminant function analysis to guide selection of a subset of other volatiles that maximised separation between the four MUP types. Known male pheromone ligands and a small set of other volatiles led to complete separation of the four MUP types in a discriminant function analysis (Fig. 5B, Table 1). This is consistent with MUP type correlating with a small subset of urinary volatiles. However, this is not evidence that mice use these volatiles to recognise MUP types. Exploration of the dataset (see Additional file 1) showed that other small subsets of volatiles could also provide separation of MUP types in this very small sample. A much larger set of independent samples would be needed to identify volatiles that consistently vary with MUP type rather than with other background variation.

**Table 1:** Standardised discriminant function coefficients for data in Fig. 5B

Retention time (min)	Tentative identity	% peak area range^a^	Standardised discriminant function coefficients		
			Function 1 87% variance	Function 2 10% variance	Function 3 3% variance
15.30	DHB	6.74–14.53	1.03	0.89	0.57
16.61	SB2HT	0.01–9.37	−0.70	−1.29	−0.09
22.32	βF	0.44–2.79	−0.72	−0.66	− 0.47
9.13	MW142	0–0.05	0.98	−1.28	−0.45
21.68	X6 (unidentified)	0.08–0.53	0.79	0.03	1.25
22.13	Acetamide	0–0.55	2.07	−0.42	−0.18
25.62	Dimethylsulphone	1.39–2.54	1.89	1.26	0.74
27.17	Phenol	0.07–0.38	0.87	1.50	0.15

^a^Peak area expressed as percentage of total volatiles; full data shown in Additional file 1

#### Darcin slows the release of male urinary signalling volatiles

**Fig. 6:**
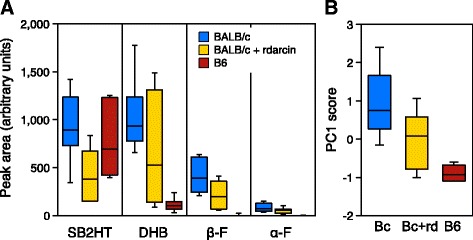
Darcin influences the release of male signalling volatiles from urine of laboratory strains. The release of 2-*sec*-butyl-4,5-dihydrothiazole (*SB2HT*), (*R,R*)-3,4-dehydro-*exo*-brevicomin (*DHB*), *E,E*-α-farnesene (*αF*) and *E*-β-farnesene (*βF*) was compared between urine from BALB/c males, B6 males and BALB/c male urine with r-darcin added to equalize that in B6 male urine (1 μg/μl). Volatiles were extracted from 10 μl urine streaked onto filter paper by HS-SPME for 10 min starting 10 min after deposition (*n* = 7 replicates per urine type) followed by GC-MS analysis. **a** Peak areas for each volatile according to urine type (boxplots show medians, interquartile and total range). **b** Principal component analysis derived a single component that accounted for strong correlation in the amount of DHB, βF and αF (76% of variance), with scores differing significantly between urine types (*F*_2,18_ = 12.71, *p* < 0.001). Bonferroni post hoc comparisons assess differences between each urine type (see text)

As females generalised between volatile signatures from different laboratory strains when the amount of darcin was artificially equalised, we compared volatiles released from B6 male urine, BALB/c male urine and BALB/c male urine with r-darcin added at approximately 1 μg/μl to match that in B6 urine. We focused on the four VOCs that are ligands of MUPs and also male pheromones that have established reproductive priming effects on female mice (SB2HT, DHB, βF, αF). Urine was streaked out to replicate behaviour tests and released volatiles were sampled by HS-SPME (*n* = 7 replicates for each of the three urine types, Fig. 6a). Principal component analysis derived a single component (PC1) that explained 76% of the variance between samples and took into account strong correlation between the amounts of DHB, αF and βF (PC1 weightings of 0.93, 0.98 and 0.98 to each volatile respectively, compared to 0.50 weighting for SB2HT). PC1 scores differed strongly between the three urine types (*F*_2,18_ = 12.71, *p* < 0.001; Fig. 6b), due to much stronger release of DHB, αF and βF from BALB/c than from B6 urine (Fig. 6a, b). Addition of r-darcin to BALB/c urine reduced emission of all three VOCs (Fig. 6a), resulting in PC1 scores that were intermediate between the two strains but differed significantly from BALB/c urine (*p* = 0.047) and not from B6 urine (*p* = 0.09, Fig. 6b). Emission of SB2HT was also reduced (*p* = 0.03, Fig. 6a), resulting in a significant difference between the three urine types (*F*_2,18_ = 4.15, *p* = 0.03). However, neither manipulated nor unmanipulated BALB/c urine differed significantly in SB2HT release from B6 male urine (*p* ≥ 0.23), due to the relatively high variation in SB2HT levels between samples from both strains (Fig. 6a). Thus, darcin appears to bind all four urinary VOCs, delaying release from urine scent marks and altering the volatile odour signature. Once r-darcin was added to BALB/c urine, release rates did not differ significantly from that of B6 urine, correlating with our behavioural findings that females generalised between these urine stimuli (Fig. 2D, E). Darcin may also influence the release of other VOCs that were not assessed here but might contribute to volatile signature recognition.

#### Darcin and central MUPs have different influences on urinary volatiles

**Fig. 7:**
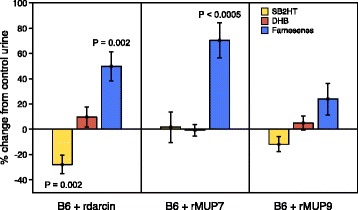
Darcin and MUP7 alter the release pattern of urinary volatiles from B6 male urine. Recombinant MUP (r-darcin, r-MUP7 or r-MUP9, 1 μg/μl) or a buffer control was added to a standard pool of B6 male urine. Volatiles were extracted from 10 μl urine streaked onto filter paper by HS-SPME for 10 min starting 10 min after deposition (*n* = 10 replicates per urine type) followed by GC-MS analysis. Data are plotted as % change from matched control urine sample (means ± SEM). *p* values indicate significant differences to control urine (planned contrasts) after MANOVA confirmed a highly significant effect of treatment on the volatiles released (*F*_9,105_ = 4.32, *p* < 0.0001). βF and αF (*farnesenes*) were summed for analysis, as both were very low in B6 urine (see Fig. 6). Planned contrasts indicated significant differences between urine types for SB2HT (*F*_3,35_ = 5.66, *p* = 0.003) and farnesenes (*F*_3,35_ = 6.53, *p* = 0.001) but not for DHB (*F*_3,35_ = 1.03, *p* = 0.39)

To compare the effects of darcin and central MUPs with different binding cavities (MUP7, MUP9) on urinary volatile signatures, we measured the same four male VOCs released when each recombinant MUP (1 μg/μl) or control buffer alone was added to a pool of B6 male urine (*n* = 10 replicates of each manipulation). As B6 male urine already contains each of these MUPs (see Ref. [44] for quantification), manipulation changed the amount rather than the presence/absence of each protein. Overall, recombinant MUP treatment had a highly significant effect on the release of these VOCs from urine streaks (multivariate analysis of variance (MANOVA), effect of treatment: *F*_9,105_ = 4.32, *p* < 0.0001). Addition of recombinant MUPs altered the release of SB2HT (*F*_3,35_ = 5.66, *p* = 0.003) and total farnesenes (βF and αF summed, *F*_3,35_ = 6.53, *p* = 0.001), but had no significant effect on the level of DHB (*F*_3,35_ = 1.03, *p* = 0.39). Each recombinant MUP had different effects. Compared to control B6 male urine with only buffer added, increased darcin reduced emission of SB2HT (*p* = 0.002), but emission of farnesenes increased (*p* = 0.002, Fig. 7). This increase in farnesene emission when darcin was increased in B6 male urine differed from the decrease observed when darcin was added to BALB/c urine (Fig. 6). This most likely reflects the very low level of farnesenes in B6 urine and the balance of two different effects of MUP binding on volatile levels: acting to increase solubility of these waxy VOCs at low levels when added to wet urine whilst reducing release rates in deposited scents. Increased MUP7 also increased farnesene emission, by an average of approximately 70% (*p* < 0.0005), but had no effect on SB2HT release (*p* = 0.67, Fig. 7). By contrast, increased MUP9 did not alter release of any of the male VOCs measured significantly (Fig. 7). These effects of manipulating MUP levels on volatile release are consistent with our behavioural findings that individual MUP profiles, including the relative amounts of male-specific darcin and MUP7, strongly influence the volatile odour signature that mice learn.

## Discussion

For rapid social recognition, animals need odours that reliably define their identity, regardless of the many other factors that influence their volatile scents. Here, we show that house mice learn attraction to a specific volatile signature that is shaped by the profile of polymorphic MUPs in a male’s scent mark. Lack of distinction between volatile signatures from males with the same MUP genotype, despite differences in total urinary VOC profiles between these males or manipulation of unbound VOCs, indicates that the male’s genetically determined MUP pattern has a dominant influence on the signature that is learned. Moreover, our ability to instigate generalisation between previously distinct volatile signatures when MUP profiles were made very similar using recombinant proteins, or to disrupt recognition when MUP profiles were altered, directly confirms that the qualitative and quantitative profile of MUPs determines this learned volatile signature. Notably, although the darcin pheromone is responsible for inducing learned attraction to a male’s odour signature [30, 32], attraction to a male’s odour did not simply increase with increasing darcin levels but instead depended on whether the amount of darcin matched that in the template that females learned on original contact with the male’s scent.

We propose that the mechanism that links MUP profile to a distinctive volatile signature is the differential binding and release of VOCs according to sequence differences that influence the conformation or availability of the MUP central cavity, binding interactions of VOCs with cavity residues, and possibly also the accessibility of the cavity [36, 41]. Consistent with this, darcin differs from other MUPs at many cavity residues predicted to influence ligand binding [28, 40], and altering the relative amount of darcin both changed recognition of the learned volatile signature and resulted in measurable differences in the release of targeted VOCs. The relative amount of central MUPs with one of two known cavity variants (phenylalanine or valine at mature sequence residue 56) also influenced volatile signatures, variants that are known to influence ligand binding [36, 39]. Whilst the phenylalanine cavity variant is more common among known central MUPs, four central *Mup* genes in the C57BL/6 reference genome encode the valine variant (*Mup7*, *Mup8*, *Mup13*, *Mup17*). Among males of common laboratory strains, MUP7 is the only valine variant expressed at detectable levels in urine [21, 52, 53]. However, MUP7, MUP8, MUP17 and two other MUPs with novel sequences but the same valine cavity variant were observed among wild male house mice derived from Edmonton, Canada, in the only study so far to identify all central MUP sequences expressed among a sample of wild mice [23]. As both cavity variants occur in multiple central MUP isoforms, and each isoform is differentially expressed according to MUP genotype, the ratio of these two central MUP cavity variants will differ between genotypes. Further, as darcin and other peripheral MUPs expressed via urine such as MUP3 and MUP21 each have different groups of cavity residues [41], variation in the ratio of each of these MUPs also increases the capacity for variation in volatile signatures defined by MUP profiles.

The specific set of VOCs that contribute to individual volatile signatures remains to be determined, as MUPs can bind a wide range of VOCs in mouse urine. Females could specifically learn volatile ligands that are associated with males (such as the four VOC pheromones assessed in this study) or incorporate a wider range of non-sex-specific volatiles that bind to MUPs [38]. Under competitive conditions, volatile signatures are particularly important for highlighting fresh scents compared to older competitor scents nearby. This results in female learned attraction to the owner of countermarks that have been deposited most recently [26, 60], and the ability to recognise territory owners at a distance by matching airborne odours from males with recent scent marks in the local area [29, 61]. However, the similarity of learned odours between males with the same MUP genotype explains why female house mice fail to discriminate when they encounter competing males that have the same MUP signature, despite other genetic differences between them [26]. Positive selection on high quality males to ensure that ownership of scent marks used to advertise their competitive ability is readily recognised is predicted to drive the evolution of such individually distinctive identity signals [62]. If the familiar volatile odour that has been learned changes (for example, due to changed status, physiology or diet), this typically stimulates further close contact investigation of the altered scent; this contact will allow the volatile signature and other volatile information to be updated whilst animals simultaneously detect the profile of MUPs directly through vomeronasal receptors [27, 31]. The stability of the MUP profile may act to buffer short-term changes caused by changes in diet, metabolome or microbiome.

Consistent with an evolved signal to reliably advertise identity, the profile of central MUP isoforms expressed by *M. m. domesticus* is individually distinctive and stable [23] (authors’ additional unpublished data) and strongly determined by MUP genotype [24, 46], as we confirm further in this study. The central array of *Mup* genes in house mice has expanded through a process of concerted evolution (non-allelic homologous recombination and gene conversion events), potentially matching a requirement for more reliable recognition of many individuals as house mouse population densities increased through exploitation of concentrated human resources [21, 63]. Sequence data indicate that frequency-dependent selection maintains diversity in both central *Mup* genes and in regulatory sequences that strongly promote individually distinctive profiles through differential transcription and gene silencing [23]. The central MUPs themselves retain 97–99% sequence identity, and most of the diversity is limited to specific surface residues, most likely by interactions with vomeronasal pheromone receptors required for direct perception of these proteins [23]. Within the binding cavity, diversity is also likely to be limited by the specific ligand binding characteristics required to provide discriminable volatile signatures. Individuality is achieved through combinatorial variation in expression of a limited set of MUPs and their relative levels, with balancing selection maintaining central MUPs at moderate frequencies in the population [23]. Investment in the peripheral MUP darcin may also be relatively stable within individual MUP profiles, though this is less well established at present. Densitometry estimated that the proportion of darcin was stable within individuals before and during competitive experience, even though total MUP output increased [51]. Our finding that females generalise between volatile signatures from males of the same MUP type also suggests that darcin levels are similar within genotypes, at least at a level that has an impact on volatile signatures.

From a theoretical perspective, components of identity signals are predicted to assort independently to promote individual diversity [2]. By contrast, MUPs are encoded by a single very tightly linked gene cluster [21, 22, 46]. However, the mechanism of differential expression that mice have evolved provides considerable capacity for distinctive signals based on the relative levels of component MUPs [23]. Indeed, tight linkage between genes contributing to this profile may be important for maintaining the stability of MUP signatures when investment in MUP output changes. A consequence of linkage is that closely related animals can inherit the same pair of MUP haplotypes, providing a distinctive identity signal when MUP genotypes differ but a signal of very close kinship when they are the same. Among competitive male house mice in outbred populations, frequent multiple mating of dams with different males [64], dispersal of males from natal territories [65] and spatial dispersion of dominant male territory owners [66, 67] should ensure that shared signatures will be uncommon between local territory owners advertising dominance through scent. By contrast, breeding female mice typically remain closely associated in kin groups [68, 69], often preferring to nest and rear offspring communally with close relatives. Females can recognise relatives through general similarity of odours, which increases with relatedness [14, 70], as we have confirmed in this study. However, female preference for related nest partners of shared MUP type when these are available indicates that shared MUP signatures are important signals of close kinship among females [46]. Avoidance of inbreeding when mice of the opposite sex share MUP signatures [71] also is evidence that MUP signatures can provide a signal of individual and/or kinship identity depending on the social context.

Typical of intraspecific communication signals, the MUP signatures evolved by house mice are highly species-specific. A recent set of studies questioned whether MUP patterns are sufficiently stable and polymorphic to provide an individual identity signal in the very closely related *Mus musculus musculus* subspecies [72–74]. Whilst this is possible, the analytical approaches used were not appropriate to resolve the small sequence differences that define each central MUP isoform and therefore could not quantify their combinatorial diversity, particularly among wild mice with unknown gene sequences (for further discussion of this, see [23, 44, 50]). A recent comparison of central MUP expression between *M. m. domesticus* and *Mus musculus musculus* subspecies in allopatry and in secondary contact revealed distinct MUP signatures between these subspecies, though both subspecies expressed the male darcin pheromone and appeared to have a similar level of complexity of central MUP isoforms [50]. Other muroid rodents express lipocalin proteins in urine used for scent communication, although those expressed by voles and hamsters (cricetid rodents) are more closely related to olfactory binding proteins (OBPs) than to MUPs [75]. The structures and central cavities of urinary lipocalin proteins are very similar across species [75–78], suggesting that all are likely to bind and release LMW hydrophobic ligands, but functional and mechanistic studies of the roles of these proteins in communication in other species are currently lacking.

Nonetheless, our findings do not mean that other odour cues play no role in social recognition in other contexts. Further, other odour differences between animals may allow simpler discriminations between classes of animals, such as ‘familiar’ versus ‘unfamiliar’ or ‘group member’ versus ‘non-group member’ rather than individual recognition per se [1]. In the context of territorial defence, for example, simple recognition of unfamiliar odours may be sufficient to allow rapid identification of unfamiliar foreign intruders without the need to recognise individual identities. Pregnancy failure, known as the Bruce effect, occurs when female mice in early pregnancy are exposed to odour from an unfamiliar male at the same time as twice-daily surges in their prolactin levels. This is stimulated by differences in LMW urinary components that include MHC peptides [79, 80], or by differences in the amount of exocrine gland-secreting peptide 1 in male tear fluids [81], compared to the remembered stud male. As females respond to particular genotype combinations among wild-stock males rather than to unfamiliar individual odour more generally [82], this appears to be due to recognition of specific differences between familiar stud male and unfamiliar foreign male odours rather than to either individual recognition or to a simple difference in familiarity.

## Conclusions

Mice identify individual scent owners using volatile odour signatures learned on contact with urine scent marks. Many genetic and physiological factors influence the total pool of volatile metabolites and pheromones in rodent urine, but we have shown that the profile of a specialised set of polymorphic binding proteins (MUPs) secreted in urine has a dominant influence on the volatile odour signatures that mice learn and use subsequently to recognise odour from the same individual. Thus, polymorphic MUP signatures provide a distinctive identity signal that can be discriminated both through the proteins themselves on nasal contact with scent and through a distinctive airborne odour signature produced by MUP-specific and volatile-specific differences in the binding and release of VOCs. Failure to distinguish between odours of genetically distinct animals that have very similar MUP profiles indicates a strong reliance on MUP signatures for individual odour recognition. This implies that variation in urine odour cues that arises incidentally from multiple differences across the genome is not sufficient to provide reliable information on the signaller’s identity in outbred mice, and we should not assume that complex body odours inevitably allow individuals to be recognised. However, when the benefits of being recognised outweigh any costs, we might expect signallers to evolve individually distinctive signals that are easy for others to recognise, as we have found in mice. Indeed, within the complex body odours of animals that derive from many different sources, specific signals of identity may be more common than previously has been realised. Our findings call for further research to establish whether investment in urinary lipocalins and their volatile ligands play similar roles in signalling genetic identity at an individual, kinship and/or species level in other rodents, and to investigate whether alternative mechanisms have evolved to signal identity reliably through scent in other species. Understanding the specific mechanisms that underlie identity recognition is likely to provide insight into constraints on social recognition across different species as well as help to explain the functions and evolution of phenotypic diversity within species.

## Methods

### Subjects and urine donors

Subjects were 453 captive bred adult female *Mus musculus domesticus* (F0–F4) aged 7–20 months, from a colony derived from wild ancestors captured from five locations in the northwest of England, UK. Most females (81.2%) were used for a single test over the total of 542 trials, but a small number were used in two (18.1%) or three (0.7%) tests, each involving different scent stimuli, with at least 2 weeks between successive tests. Females were housed in 45 × 28 × 13 cm cages (MB1, North Kent Plastics, Leicestershire, UK) in single-sex small family groups (two to four sisters per cage during the test period) in a different room from the urine donors. As we have previously shown that females show very similar attraction responses to urine stimuli from unfamiliar males whether completely naïve to adult male scents or after natural social and sexual experience [42], subjects were not isolated from contact with other adult male scents in the animal unit, and soiled bedding from captive bred wild males was regularly added to female cages during the testing period to ensure normal oestrus cycling.

Urine donors were 21 male and 15 female C57BL/6 laboratory mice (C57BL/6OlaHsd, Harlan, UK) aged 4–12 months, 18 male and 17 female BALB/c mice (BALB/cOlaHsd, Harlan, UK) aged 4–12 months, 8 male and 10 female CD-1 mice (Hsd:ICR(CD-1®), Harlan, UK) aged 4–12 months and 72 captive bred adult male house mice derived from the same colony as the female subjects but unrelated. Males were housed singly in 43 × 11.5 × 12 cm cages (M3, North Kent Plastics) to ensure that there were no subordinate males whose scent is unattractive to females. Females were housed in single-sex small groups (two to four) in 45 × 28 × 13 cm cages. Urine was collected by holding the donor mouse by the scruff of the neck over a clean 1.5-ml microtube. In tests of response to wild-stock male odours, female replicates were each tested with urine from different individual males (assigned randomly within the constraint of no prior familiarity). Urine from laboratory strains was pooled from five to eight individual donors of the same strain and sex for testing, using different combinations of donors to create each stimulus pool. Pooled urine from inbred BALB/c strain females was used in most tests as a standard female control stimulus that differed from the subject’s own scent. In tests using BALB/c male urine as the stimulus, C57BL/6 female urine provided the female control stimulus. Urine was collected up to 1–2 weeks prior to testing and stored at –20 °C until use.

Throughout, all animals were housed on a reversed 12:12 h light cycle with lights off at 08:00 h, and were maintained on Corn Cob Absorb 10/14 substrate with paper wool nest material and ad libitum access to water and food (5LF2 Certified Eurodiet 14% in the final experiment shown in Fig. 2E, otherwise Lab Diet 5002 Certified Rodent Diet, Purina Mills, St Louis, MO, USA). Cardboard tubes and red plastic mouse houses (Techniplast) were provided for home cage enrichment. Animals were transferred between cages and test arenas using a handling tunnel to minimise any stress [83].

### Behavioural recognition assay

Three days prior to each trial, soiled nest material and substrate from wild-derived males (different from test stimulus donors) was introduced into the subject female’s home cage to induce oestrus during the preference test. Previous studies using this procedure on mice in our colony has shown that over 95% of females were likely to be in oestrus or pro-oestrus during a test, and would show a robust attraction to a male urine stimulus compared to an equivalent female stimulus when allowed to contact the urine stimuli [26, 32, 42]. All testing was carried out during the dark phase of the light cycle under dim red lighting.

Tests followed the procedure described in Ref. [32]. For all tests, females had prior contact with a male urinary test stimulus as a learning template that contained the darcin pheromone, which induces learned attraction to the volatile signature of the male through associative learning [30, 32]. Individual females were given contact with the male learning template (10 μl of male urine with or without the addition of a recombinant MUP or buffer) and a female control stimulus (female urine with or without buffer to match the male learning template) for 30 min in a clean arena (45 × 28 × 13 cm). Each contact stimulus was streaked onto GF/C glass micro-fibre filters (55 mm diameter cut in half, Whatman, Maidstone, UK), held outside mesh spheres that allowed full nasal contact with the stimuli. Females were then transferred to a clean test arena (45 × 28 × 13 cm MB1 cage base fitted with a Perspex lid perforated by 6-mm holes evenly distributed 20 mm apart) to which females were familiarised for 30 min immediately before each 10-min test. A fresh male test stimulus and the female control stimulus (10 μl) were streaked onto glass microfibre filters as described above, allowed to dry for approximately 10 min and then suspended 2 cm above the perforated Perspex arena lid using a 5-cm diameter Perspex cylinder to ensure that the mice could not physically contact the test stimuli during the test. Test stimuli were placed 25 cm apart in the central zone of the test arena, where wild mice normally spend little time, so that increased time in these sites reflected attraction to the scent. Note that we used female urine as a control novel urine stimulus in the recognition assay (present in both the learning and test phases) rather than presenting subjects with a choice between two different male odours. This avoids conflating the ability to recognise individual males with potential differences in their quality, genetic compatibility and simple odour familiarity from the learning phase that could also influence relative female attraction.

Females were assigned randomly to a particular test stimulus in each series of tests conducted, and trials within a test series were run in random order. The location of the male odour was also assigned at random to side A or B. Behaviour was transcribed from DVD recordings blind to both the position of each odour and the specific odour stimulus within each series of tests. We recorded time under each stimulus (nose within the 55-mm diameter circle in which a test stimulus was presented) when the animal was sniffing or not sniffing at the stimulus. Recognition was assessed as greater total time spent under the male compared to the female control stimulus. The sample size for most tests was *n* = 12 replicates. This was raised to *n* = 20 replicates for some tests where we initially predicted that recognition might be more difficult (tests shown in Fig. 1D (f–i)) or to increase statistical power to draw a clear conclusion (Fig. 2C (e), d (g, i, j, l)). A small number of trials (*n* = 8 or 1.5%) had to be dropped from analyses because the female sat in a corner of the arena throughout the test (1/12 in test Fig. 1B (a), 3/20 in test Fig. 1D (f), 1/20 in test Fig. 2D (j), 1/12 in test Fig. 2E (r)) or showed stereotypical behaviour (2/20 in test Fig. 2D (j)). Full datasets and sample sizes are given in Additional file 2.

### Fractionation and recombination of urine

Male urine was separated into high molecular weight (HMW) and low molecular weight components (LMW) using Vivaspin centrifugal concentrators (cut-off 3 kDa, Sartorius UK Ltd., Epsom, UK). The filters were first washed with water twice, after which a natural or manipulated urine sample was added to the concentrator and centrifuged for 30 min at 15,000 g. The material passing through the filter was referred to as ‘LMW’; that retained by the filter was referred to as ‘HMW’. HMW and LMW were recombined in original proportions.

### Manipulation of male urine with recombinant MUPs

Recombinant MUPs (r-darcin, r-MUP7, r-MUP9) were prepared as described in Ref. [32]. To estimate the amount of natural darcin in urine stimuli from laboratory strain males, urine was pooled from five to eight males of the same strain and the total protein concentration of each sample measured using the Coomassie Plus® protein assay (Pierce, Rockford, IL, USA). A fixed amount of protein (0.25 μg and 0.125 μg) per sample was applied to sodium dodecyl sulphate-polyacrylamide gel electrophoresis (SDS-PAGE) analysis according to Ref. [84]. Gels were scanned using an Epson Perfection V750 Pro scanner, and the proportion of protein in the main MUP band and the darcin band [40] was quantified by densitometry using TotalLab Quant software (Nonlinear Dynamics Ltd., Newcastle upon Tyne, UK). The amount of darcin was calculated as the total protein concentration multiplied by the proportional volume of the darcin band (averaged over the two concentrations run per sample) compared to the MUP band. This was used to calculate the amount of r-darcin needed to equalise darcin concentrations between the particular urine samples used in each test. In all test series where r-MUP was added to a test stimulus, an equivalent volume of buffer was added to the female control stimulus and to any other urine samples being compared. A standard amount of 1 μg/μl r-MUP was used in all other tests (matching the approximate amount of r-darcin in C57BL/6 male urine).

### Analysis of volatiles emitted from urinary scent marks

Urine (10 μl) was applied to a strip of GF/C glass micro-fibre filter (Whatman) suspended on a pin. As many urinary metabolites are lost through evaporation over the first few minutes after urine deposition [85] and thus do not contribute to longer term information held in scent marks, urine samples were dried for 10 min at ambient temperature (approximately 20 °C) to match our behavioural tests before the strip of filter was placed in a 6-ml headspace vial and sealed. The volatiles were extracted by SPME for 10 min at ambient temperature using a 1-cm divinylbenzene (DVB)/Carboxen (CAR)/polydimethylsiloxane (PDMS) fibre (Supelco, Bellefonte, PA, USA). The extracts were then analysed by gas chromatography-mass spectrometry (GC-MS) using a Waters GCT mass spectrometer (Waters, Wilmslow, UK). The GC column used was a 30 m × 0.2 mm internal diameter (i.d.), 20-μm film thickness Supelcowax 10 (Supelco). The GC inlet was configured in the splitless mode, and the injector temperature was 220 °C. The GC oven temperature programme was 50 °C to 220 °C at 6 °C/min; the temperature was then held at 220 °C for 8 min. The same SPME fibre was used for all extractions and was conditioned before each use in a Field Forensics CN303R conditioner (Field Forensics, St Petersburg, Florida, USA). The mass spectrometer scan range was *m/z* 50 to 300, and the scan time was 0.5 s. GC-MS peak areas for 3,4-dehydro-brevicomin, 2-*sec*-butyl-4,5-dihydrothiazole, *E,E*-α-farnesene and *E*-β-farnesene were obtained from summed ion chromatograms produced in MassLynx software (Waters). All data were averaged technical duplicates. Urine samples were run in randomised order and analysed blind to treatment identity.

To compare volatiles emitted from C57BL/6 male urine, BALB/c male urine and BALB/c urine with r-darcin added to equal the concentration in C57BL/6 urine, seven separate urine pools were collected for each treatment. Recombinant darcin was added to pools of the third treatment group to match that in C57BL/6 male urine, and all urine samples were frozen at –20 °C until VOC analysis. To compare the effect of increasing the amount of darcin, MUP7 and MUP9 in male urine on volatile release from urine scent marks, a single pool of C57BL/6 male urine was split into 40 separate aliquots and 1 μg/μl of r-darcin, r-MUP7, r-MUP9 or an equivalent volume of buffer was added to 10 replicate aliquots each. Samples were frozen at –20 °C until VOC analysis.

### MUP and MHC genotypes

MUP and MHC haplotypes carried by stimulus males were established by genotyping the males themselves, their parents and grandparents using microsatellite markers on either side of the MUP and MHC gene clusters to ensure that MUP or MHC types defined as the same were genetically identical through recent common descent (see Ref. [46] for full details).

### Electrospray ionisation mass spectrometry

Urine samples were diluted 1:100 in 0.1% (v/v) formic acid without any pretreatment. Analyses were performed on a Nano Acquity Ultra Performance Liquid Chromatography (UPLC) system (Waters) coupled to a Q-ToF micro mass spectrometer (Waters), fitted with an ESI source. The calibrant was a 2 pmol/μl solution of horse heart myoglobin (Sigma, Munich, Germany) in 2 mM dithiothreitol (DTT, Melford Laboratories, Ipswich, UK) 0.1% (v/v) formic acid (Sigma-Aldrich, Gillingham, UK). Samples (6 μl) were desalted and concentrated on a C4 reverse phase trap (LC Packings, Amsterdam, the Netherlands). MUPs were eluted at a flow rate of 40 μl/min using repeated 5–95% acetonitrile gradients. High-performance liquid chromatography (HPLC) grade water (VWR International, Lutterworth, UK) and acetonitrile (Fisher Scientific, Loughborough, UK) were used. The mass spectrometer scan range was *m/z* 800 and 1600. The multiply charged mass spectra were processed and transformed to a true mass scale using MaxENT 1 maximum entropy software (Waters). All datasets were processed at 0.25 Da/channel over a mass range of 18,400–19,000 Da, and a peak width of 0.75 Da was used to construct the damage model. This method separates MUPs in each urine sample that differ in mass by 1–2 Da or more and provides sufficient resolution to show the very strong similarity in central MUP profiles between males with the same MUP genotype and different profiles in those of different MUP genotype.

### Data analysis

All data exploration and analyses were carried out using IBM SPSS Statistics version 24. We assessed recognition of male volatile signatures in behavioural assays as greater total time spent under the male compared to the female control stimulus, using matched-pair *t* tests (log transformed to meet assumptions of parametric analysis if appropriate) or non-parametric Wilcoxon matched-pair tests where data could not be transformed to meet parametric assumptions (data for all behavioural recognition tests are provided in Additional file 2). The distribution of behavioural responses (bias in time near male minus time near control female stimulus) was compared between tests when odour was from the same male versus from a male of the same MUP type or different MUP type using Kolmogorov-Smirnov tests.

To examine similarity in MUP profiles between samples, correlation coefficients between pairs of samples were calculated based on the area of seven mass peaks differentiated in the intact mass profiles of the males sampled (mass peaks 18,645 Da, 18,666 Da, 18,682 Da, 18,693 Da, 18,709 Da, 18,713 Da, 18,893 Da) [23]. Each of these masses corresponds to one or more known MUPs. Pearson correlation coefficients were also calculated based on the proportional areas of 134 VOC peaks detected by SPME extraction of urine samples and GC-MS analysis (data provided in Additional file 1). Analyses of variance (ANOVAs) compared the effects of MUP sharing and relatedness between males on these correlation coefficients. Discriminant function analysis was used to explore whether a subset of volatile peaks would separate MUP types in a small set of samples from wild-stock males, although the sample size was too small for meaningful statistical analysis.

To compare relative levels of four male VOCs between BALB/c male urine, C57BL/6 male urine and BALB/c male urine with added r-darcin, principal component analysis was used to derive a single component that took into account very strong correlation in the amount of DHB, αF and βF between samples. ANOVAs compared scores for this component, or the amount of SB2HT, followed by Bonferroni post hoc comparisons between each urine type (data provided in Additional file 3). The overall effect of adding different recombinant MUPs to a pool of C57BL/6 male urine on the emission of SB2HT, DHB and summed αF + βF was assessed by MANOVA, whilst planned comparisons examined the effects of recombinant manipulation on each VOC separately (data provided in Additional file 4).

## Additional files

Additional file 1: Intact mass spectra and GC-MS data from trios of wild male brothers used for Figs. 4 and 5. Figure 4AB tab provides intact mass spectra (expressed as proportion of highest peak) for each male according to sib group and genotype. Figure 4CD tab provides areas for 134 volatiles peaks (expressed as % total peak area) and principal component analysis. (XLS 232 kb)

Additional file 2: Datasets from behavioural recognition tests in Figs. 1, 2 and 3. Time spent under male and female stimuli for each test and replicate together with summary data and statistical comparison. (XLS 67 kb)

Additional file 3: GC-MS analysis used for Fig. 6. Peak areas and principal component scores for the four male-specific volatiles measured from BALB/c urine, C57BL/6 urine and BALB/c urine + r-darcin. (XLS 31 kb)

Additional file 4: GC-MS analysis used for Fig. 7. Peak areas for the four male-specific volatiles measured from a pool of C57BL/6 urine when different recombinant MUPs or buffer only was added, together with duration of storage prior to analysis. (XLS 32 kb)

## Abbreviations

βF: *E*-β-farnesene
αF: *E,E*-α-farnesene
B6: C57BL/6 strain of mice
DHB: (*R,R*)-3,4-dehydro-*exo*-brevicomin
HMW: High molecular weight
HS-SPME: Headspace solid phase micro-extraction
LMW: Low molecular weight
MHC: Major histocompatibility complex
MUP: Major urinary protein
SB2HT: 2-*sec*-butyl-4,5-dihydrothiazole
VOC: Volatile organic compound
